# Disruption of the Cx43/miR21 pathway leads to osteocyte apoptosis and increased osteoclastogenesis with aging

**DOI:** 10.1111/acel.12586

**Published:** 2017-03-19

**Authors:** Hannah M. Davis, Rafael Pacheco‐Costa, Emily G. Atkinson, Lucas R. Brun, Arancha R. Gortazar, Julia Harris, Masahiro Hiasa, Surajudeen A. Bolarinwa, Toshiyuki Yoneda, Mircea Ivan, Angela Bruzzaniti, Teresita Bellido, Lilian I. Plotkin

**Affiliations:** ^1^ Department of Anatomy & Cell BiologyIndiana University School of MedicineIndianapolisINUSA; ^2^ Instituto de Medicina Molecular AplicadaFacultad de MedicinaUniversidad San Pablo‐CEUMadridSpain; ^3^ Division of Hematology/OncologyDepartment of Internal MedicineIndiana University School of MedicineIndianapolisINUSA; ^4^ Department of Oral BiologyIndiana University School of DentistryIndianapolisINUSA; ^5^ Division of EndocrinologyDepartment of Internal MedicineIndiana University School of MedicineIndianapolisINUSA; ^6^ Roudebush Veterans Administration Medical CenterIndianapolisINUSA

**Keywords:** aging, apoptosis, connexin43, HMGB1, miR21, osteocyte

## Abstract

Skeletal aging results in apoptosis of osteocytes, cells embedded in bone that control the generation/function of bone forming and resorbing cells. Aging also decreases connexin43 (Cx43) expression in bone; and osteocytic Cx43 deletion partially mimics the skeletal phenotype of old mice. Particularly, aging and Cx43 deletion increase osteocyte apoptosis, and osteoclast number and bone resorption on endocortical bone surfaces. We examined herein the molecular signaling events responsible for osteocyte apoptosis and osteoclast recruitment triggered by aging and Cx43 deficiency. Cx43‐silenced MLO‐Y4 osteocytic (Cx43^def^) cells undergo spontaneous cell death in culture through caspase‐3 activation and exhibit increased levels of apoptosis‐related genes, and only transfection of Cx43 constructs able to form gap junction channels reverses Cx43^def^ cell death. Cx43^def^ cells and bones from old mice exhibit reduced levels of the pro‐survival microRNA miR21 and, consistently, increased levels of the miR21 target phosphatase and tensin homolog (PTEN) and reduced phosphorylated Akt, whereas PTEN inhibition reduces Cx43^def^ cell apoptosis. miR21 reduction is sufficient to induce apoptosis of Cx43‐expressing cells and miR21 deletion in miR21^fl/fl^ bones increases apoptosis‐related gene expression, whereas a miR21 mimic prevents Cx43^def^ cell apoptosis, demonstrating that miR21 lies downstream of Cx43. Cx43^def^ cells release more osteoclastogenic cytokines [receptor activator of NFκB ligand (RANKL)/high‐mobility group box‐1 (HMGB1)], and caspase‐3 inhibition prevents RANKL/HMGB1 release and the increased osteoclastogenesis induced by conditioned media from Cx43^def^ cells, which is blocked by antagonizing HMGB1‐RAGE interaction. These findings identify a novel Cx43/miR21/HMGB1/RANKL pathway involved in preventing osteocyte apoptosis that also controls osteoclast formation/recruitment and is impaired with aging.

## Introduction

Increasing evidence suggests that the viability of osteocytes embedded within the bone mineral matrix is essential for maintaining skeletal homeostasis, as supported by the increase in apoptotic osteocytes and prevalence of empty lacunae observed in conditions of elevated bone fragility and with old age (Plotkin & Bellido, 2016). Osteocytes have long dendritic processes that create an extensive network allowing for communication between adjacent osteocytes and with osteoblasts and osteoclasts on the bone surface, and disruption of this network impairs osteocytic regulation of bone formation and resorption (Bonewald, 2011). Osteocyte interactions with other cells occur through the release of cytokines to the lacunar–canalicular system that surrounds osteocytes, as well as through cell‐to‐cell communication via gap junction channels (Plotkin & Bellido, 2013). Previous work from our group has demonstrated that the gap junction protein connexin43 (Cx43) is a critical component of the signaling pathway controlling osteocyte survival, as evidenced by the increased osteocyte apoptosis in mice lacking Cx43 in osteoblasts and osteocytes or only in osteocytes (Plotkin *et al*., 2008; Bivi *et al*., 2012). In addition to increased osteocyte apoptosis, mice lacking osteocytic Cx43 (Cx43^ΔOt^) exhibit enhanced endocortical resorption, partially mimicking the phenotype of old mice (Almeida *et al*., 2007b) and raising the possibility that decreased Cx43 contributes at least in part to the skeletal phenotype of aging. However, while it has been shown that Cx43 levels in osteocytes are decreased in old mice (Kar *et al*., 2013), the signaling pathway activated by Cx43 deficiency involved in osteocyte apoptosis with aging is not known.

One of the potential molecules that could mediate osteocyte apoptosis is microRNAs (miRs), single‐stranded noncoding RNAs that negatively regulate gene expression (Peng *et al*., 2016). miRs are involved in cancer cell survival, and in particular, the expression of miR21 is upregulated in a variety of cancers, promoting cell survival through the direct inhibition of apoptotic genes including phosphatase and tensin homolog (PTEN) (Garzon *et al*., 2009). In bone, miRs play an important role in controlling the function and lifespan of both osteoclasts and osteoblasts (He *et al*., 2009). However, the role of miRs on osteocyte apoptosis has not been investigated.

Several models of increased osteocyte death, including ovariectomy, unloading, microdamage, and a transgenic model of osteocyte apoptosis induced by diphtheria toxin, exhibited co‐localization of apoptotic osteocytes and osteoclasts, suggesting that signals released by dying osteocytes induce osteoclast recruitment (Aguirre *et al*., 2006; Tatsumi *et al*., 2007; Cardoso *et al*., 2009; Emerton *et al*., 2009). However, the nature of these signals remains unknown. In osteocytes and other cells, apoptotic cell death results in the release of the pro‐inflammatory cytokine high‐mobility group box‐1 (HMGB1), a nonhistone nuclear DNA‐binding protein responsible for stabilization of nucleosomal structures facilitating gene transcription (Charoonpatrapong *et al*., 2006; Yang *et al*., 2008). HMGB1 released from the cells activates the receptor for advanced glycation end products (RAGE) and Toll‐like receptor 4 (TLR4) and can regulate recruitment and differentiation of osteoclast precursors (Zhou *et al*., 2008; Yang *et al*., 2013). However, whether HMGB1 is involved in osteoclast recruitment and differentiation induced by apoptotic osteocytes is not known.

Based on the results of this study, we propose that miR21 lies downstream of Cx43 in the control of osteocyte viability, and that increased osteocyte apoptosis in the absence of Cx43 and with aging is a consequence of disruptions in the PTEN/pAkt pathway and reduced response to the insulin‐like growth factor 1 (IGF‐1). Further, the increased osteoclastogenic potential of Cx43‐deficient osteocytes is a result of elevated release of the cytokines RANKL and HMGB1 during apoptosis, and the subsequent activation of RAGE on osteoclast progenitors. In conclusion, our data identify a novel Cx43/miR21/HMGB1/RANKL pathway mediated by gap junction communication in osteocytes that could be targeted to treat bone fragility in aging.

## Results

### Aging and Cx43 deficiency result in increased osteocyte apoptosis

**Figure 1:**
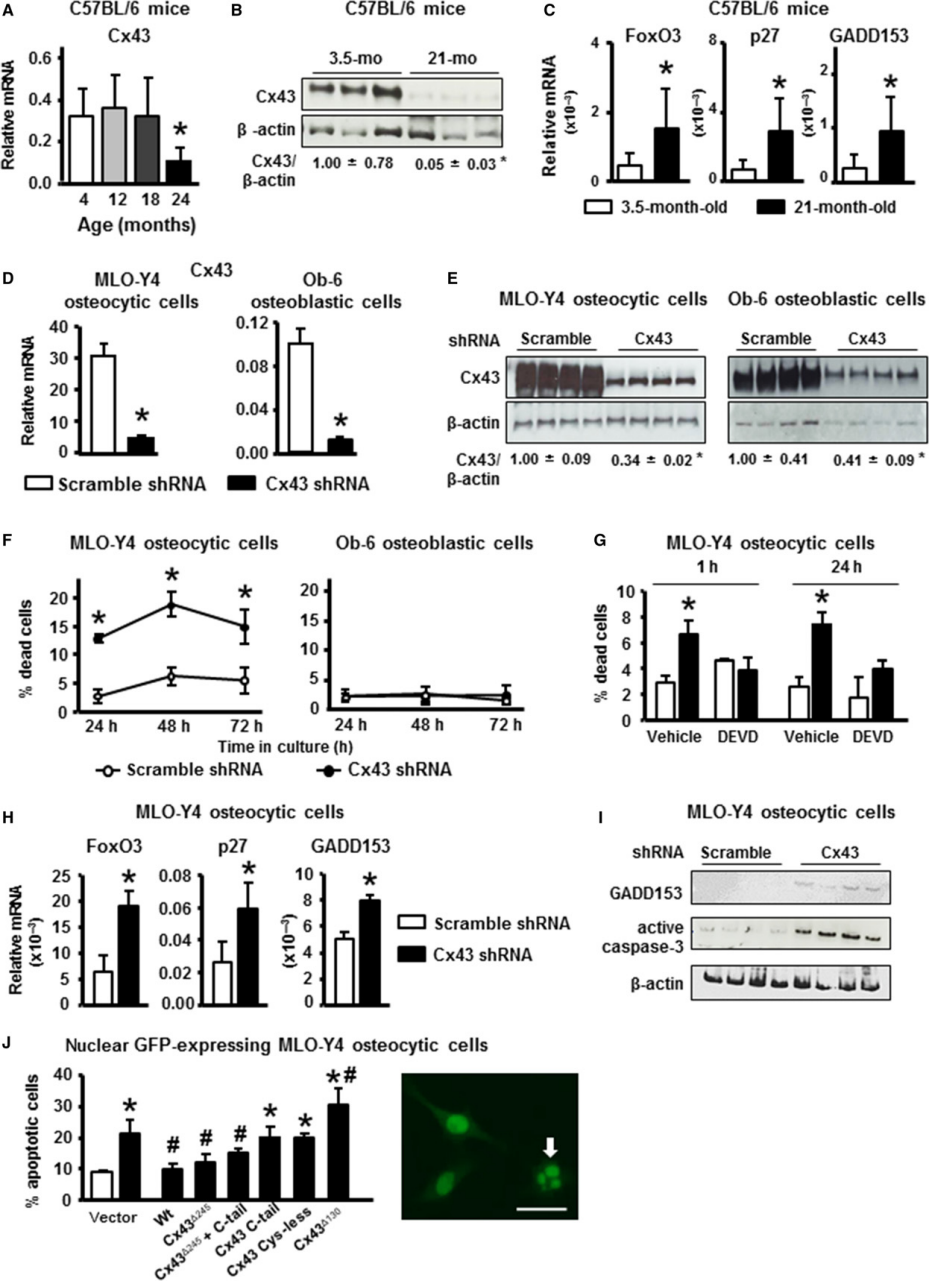
Aging and deletion of Cx43 in MLO‐Y4 osteocytic cells leads to increased apoptosis. (A) Cx43 mRNA levels in calvaria from 4‐ to 24‐month‐old mice measured by qPCR and corrected by GAPDH (Cx43 *n* = 5–9). (B) Cx43 protein levels in L5 vertebra from mice at 3.5 and 21 months of age were assessed by Western blotting and normalized by β‐actin (*n* = 6–7). (C) mRNA levels for the indicated apoptosis‐associated genes in tibia from young and old mice. (D) Cx43 protein expression was measured in MLO‐Y4 osteocytic (*n* = 6–9) and Ob‐6 (*n* = 3) expressing (scramble) or lacking (shRNA) Cx43 by qPCR and corrected by GAPDH. (E) Cx43 protein expression was measured in MLO‐Y4 osteocytic and Ob‐6 osteoblastic cells by Western blot analysis and normalized by β‐actin (*n* = 4). (F) Cell death assessed in MLO‐Y4 osteocytic cells (*n* = 3) and in Ob‐6 cells (*n* = 6) expressing or not Cx43 by Trypan blue uptake after culturing them for the indicated times. (G) MLO‐Y4 osteocytic cells silenced or not for Cx43 were treated for 1 or 24 h with DEVD and percentage of cells undergoing cell death were assessed by Trypan blue uptake (*n* = 6). (H) Gene expression was measured by qPCR and corrected by GAPDH (*n* = 6–9). (I) Protein expression for GADD153 and active caspase‐3 assessed by Western blotting (*n* = 4). (J) MLO‐Y4 osteocytic cells were transiently transfected with empty vector or the indicated Cx43 constructs together with nGFP. Apoptosis was determined by evaluating nuclear morphology of the transfected (green fluorescent) cells. A representative image of cells exhibiting nuclear fragmentation (arrow) compared to other cells with normal morphology is shown. Bars and dots represent mean ± SD. *P < 0.05 vs. 4 months of age, by one‐way ANOVA (A), *P < 0.05 vs. young (B and C) or vs. scramble shRNA cells (D‐I), by t‐test. *P < 0.05 vs. vector‐transfected scramble shRNA cells, ^#^ *P* < 0.05 vs. vector‐transfected Cx43 shRNA cells by one‐way ANOVA (J).

Bones from 24‐month‐old mice exhibited a decrease in Cx43 mRNA levels compared to 4‐month‐old mice (Fig. 1A), and 21‐month‐old mice exhibited a 95% decrease in Cx43 protein levels, compared to young, 3.5‐month‐old mice (Fig. 1B). Consistent with the increased osteocyte apoptosis reported in old humans and mice (Qiu *et al*., 2002; Almeida *et al*., 2007b), bones (without bone marrow) from 21‐month‐old mice exhibit increased expression of apoptosis‐related genes (Fig. 1C). To further study the connection between reduced Cx43 levels and osteocyte apoptosis, we used an *in vitro* system in which Cx43 was silenced in osteocytic and osteoblastic cells. Silencing Cx43 in MLO‐Y4 osteocytic and Ob‐6 osteoblastic cells using shRNA resulted in a significant reduction in mRNA levels (Fig. 1D), and ~ 70% and 60% reduction at the protein level respectively, compared to cells treated with scramble shRNA (Fig. 1E). Decreased Cx43 expression led to increased cell death in culture over time in MLO‐Y4 osteocytic cells, as previously shown (Bivi *et al*., 2012). Cx43 deficiency does not increase osteoblast apoptosis *in vivo*, and, similarly, silencing Cx43 in Ob‐6 osteoblastic cells did not alter viability, compared to scramble shRNA‐treated cells (Fig. 1F). Cell death in MLO‐Y4 osteocytic cells lacking Cx43 was reversed by DEVD treatment, an irreversible active caspase‐3 inhibitor, indicating cell death by apoptosis (Fig. 1G). Furthermore, mRNA levels of FoxO3, p27, and GADD153 genes involved with apoptosis (Zhang *et al*., 2011) were increased in MLO‐Y4 Cx43‐silenced cells (Fig. 1H) but unaltered in Ob‐6 cells (Fig. S1A). Consistent with this, protein levels for GADD153 and for the apoptosis marker active caspase‐3 were increased in Cx43‐deficient cells (Fig. 1I).

We next investigated the domain of Cx43 required to maintain cell viability. As expected, expression of full‐length Cx43 decreased apoptosis in Cx43‐silenced MLO‐Y4 cells (Figs 1J and S1B). Similarly, Cx43^Δ245^, lacking the C‐terminus scaffold domain, as well as simultaneous transfection with Cx43^Δ245^ and the C‐terminus domain (C‐tail), which reassemble to form a complete connexin molecule in the cell (Hoshi *et al*., 1990), reversed the increase in apoptosis. On the other hand, the Cx43^Δ245^ mutant is not able to confer responsiveness to bisphosphonates or to PTH (Plotkin *et al*., 2002; Bivi *et al*., 2011). Cx43‐silenced cells expressing the Cx43 C‐tail alone or Cx43^Cys‐less^, a mutant that due to the lack of cysteine residues in the extracellular domain (Dahl *et al*., 1991) only forms hemichannels and it is unable for form gap junctions (Bao *et al*., 2004; Tong *et al*., 2007), maintained an elevated percentage of apoptosis, similar to vector‐transfected cells. This contrasts with the reversal of the effect of Cx43 silencing in osteoblastic cells by the same Cx43^Cys‐less^ mutant (Bivi *et al*., 2011). While the experiment reported in Fig. 1J shows that only constructs with intact intercellular communication are able to reverse the increase in cell apoptosis in the absence of Cx43, the experiment of Fig. S1B (Supporting information) shows that expression of the mutant that can only form hemichannels (Cys‐less) and the C‐tail alone partially reduced apoptosis in Cx43‐deficient cells. This discrepancy could be due to different levels of expression of the construct in the transient transfections or to the different endpoints evaluated in each experiment. Expression of Cx43^Δ130^, a mutant that lacks channel permeability (Krutovskikh *et al*., 1998), not only did not reverse the increase in cell death, but further increased apoptosis, compared to Cx43‐silenced cells transfected by empty vector.

### Decreased Cx43 expression reduces miR21, promoting osteocyte apoptosis

**Figure 2:**
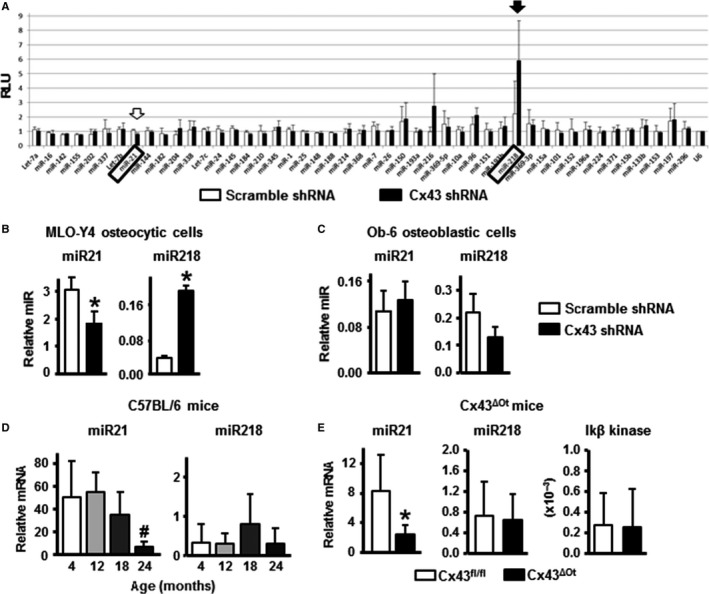
Cx43 deficiency and old age results in decreased miR21 expression. (A) Profile of 48 apoptosis‐associated miRNA assessed in MLO‐Y4 osteocytic cells expressing or not for Cx43 using an miRNA plate array and corrected by U6 RNA (*n* = 3). (B) Changes in the expression of miR21 and miR218 in MLO‐Y4 osteocytic cells were validated by qPCR and corrected by miR135 (*n* = 3). (C) Expression of miR21 and miR218 corrected by miR135 in Ob‐6 osteoblastic cells (*n* = 3). (D) miRNA expression in calvaria from 4‐ to 24‐month‐old mice measured by qPCR and corrected by miR135 (*n* = 5–9). Bars represent mean ± SD. ^#^ *P* < 0.05 vs. 12 months of age, by one‐way ANOVA. (E) Gene and miRNA expression in calvaria from Cx43^fl/fl^ and Cx43^ΔOt^ mice measured by qPCR and corrected by miR135 (miR21 and miR218) or GAPDH (Ikβ kinase B). Bars represent mean ± SD (*n* = 5–8). *P < 0.05 vs. Cx43^fl/fl^ mice, by *t*‐test.

We next examined whether increased apoptosis was associated with dysregulation of miRs in Cx43‐deficient osteocytic cells. Screening of apoptosis‐associated miRNAs revealed a decrease in the levels of miR21, which promotes survival in various cell types (Garzon *et al*., 2009) but is of unknown function in osteoblastic cells (Fig. 2A). On the contrary, miR218, a pro‐apoptotic miR, was expressed at higher levels in Cx43‐deficient MLO‐Y4 cells, compared to cells treated with scramble shRNA. Changes in the expression were confirmed by qPCR, which showed that miR21 is decreased by 50% whereas miR218 is increased by 500% in Cx43‐deficient osteocytic cells (Fig. 2B). Consistent with increases in miR218, expression of its target IκB kinase B was reduced (not shown). On the other hand, silencing Cx43 did not alter the levels of miR21, miR218 (Fig. 2C), or IκB kinase B (not shown) in Ob‐6 osteoblastic cells. In addition, 24‐month‐old mice exhibited a decrease in miR21 levels, compared to young mice, similar to Cx43^def^ cells. The expression of miR218 was not significantly changed in the bones from aging mice, although it showed a tendency toward increase in 18‐month‐old mice (Fig. 2D). Furthermore, Cx43^ΔOt^ mice lacking Cx43 in osteocytes exhibit reduced miR21 levels; however, miR218 and IκB kinase B were not altered (Fig. 2E).

### Reduced miR21 levels lead to osteocytic cell death and increased PTEN levels in bone

**Figure 3:**
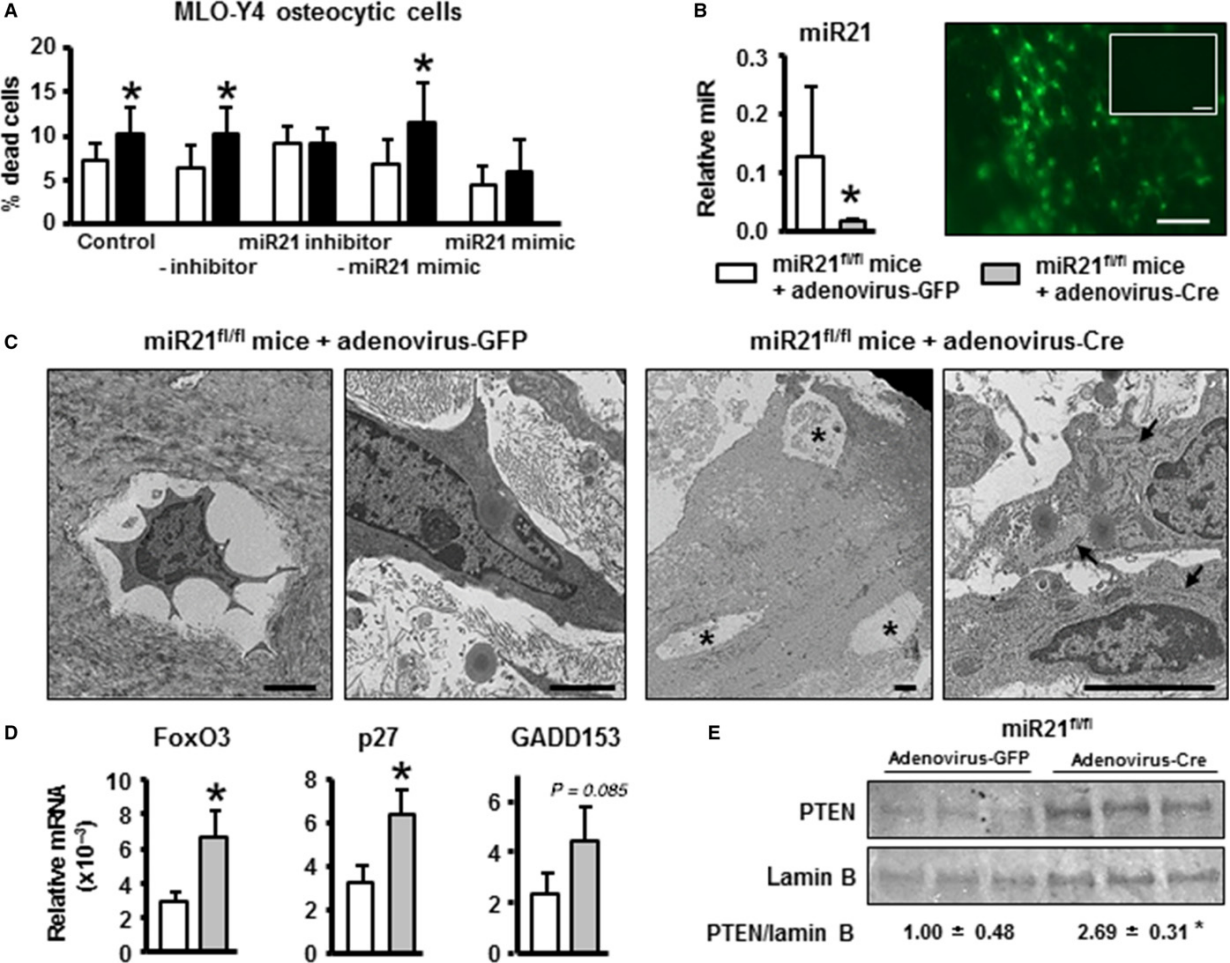
Deletion of miR21 is sufficient to induce osteocyte apoptosis and increase PTEN levels in bone. (A) MLO‐Y4 osteocytic cells were transfected with the indicated oligonucleotides, and cell death was assessed by Trypan blue uptake (*n* = 6). *P < 0.05 vs. corresponding scramble shRNA cells, by *t*‐test. (B) Neonatal calvariae from miR21^fl/fl^ mice at 5 days of age were treated with adenovirus‐Cre to delete miR21 gene. miR21 levels corrected by miR135 measured by qPCR. Representative images of viral infection efficiency under fluorescence microscope in adenovirus‐GFP calvariae and adenovirus‐Cre (without GFP expression) calvariae (inset) are shown. Scale bar indicates 100 μm. (C) TEM images of neonatal calvariae from miR21^fl/fl^ mice treated with adenovirus‐GFP (control) or adenovirus‐Cre. Images are representative of three samples. Asterisks indicate empty lacunae, and arrows point at the endoplasmic reticulum. Scale bars indicate 2 μm. (D) Gene expression measured in calvaria by qPCR and corrected by GAPDH (*n* = 3). Bars represent mean ± SD. (E) PTEN expression in calvaria was assessed 48 h after miR21 deletion by Western blotting and normalized to lamin B levels (*n* = 3). (B–E) *P < 0.05 vs. corresponding cells infected with adenovirus‐GFP, by *t*‐test.

Similar to silencing the Cx43 gene, reduction of miR21 expression by transfecting a specific oligonucleotide inhibitor induced apoptosis of control osteocytes, whereas transfection of a miR21‐mimic oligonucleotide resulted in lower levels of dead cells in scramble‐transfected cells (Fig. 3A). Further, miR21 mimic reversed the increased apoptosis of Cx43‐deficient osteocytes. To further assess the role of miR21 on bone cell apoptosis, neonatal calvaria bone from miR21^fl/fl^ mice was treated with adenovirus‐Cre to delete the miR21 gene, and compared to bone treated with adenovirus‐GFP as control (Fig. 3B–E). Addition of adenovirus‐Cre resulted in a 91% reduction of miR21 levels (Fig. 3B). Ultrastructural analysis of osteocytes using transmission electron microscopy revealed that adenovirus‐Cre‐treated calvariae from miR21^fl/fl^ mice exhibited abundant empty lacunae (Fig. 3C). Furthermore, osteocytes, when present, exhibited swollen endoplasmic reticulum, an indicator of cellular stress when compared to calvaria bones infected with adenovirus‐GFP. Similar to Cx43‐deficient cells, bones with deleted miR21 exhibited increased expression of the apoptosis‐related genes FoxO3 and p27, and a tendency toward increased GADD153 levels (Fig. 3D). Moreover, protein levels of the miR21 target phosphatase PTEN were increased by ~ 2.7‐fold (Fig. 3E).

### Cx43 deficiency and aging result in disruptions in PTEN/pAkt pathway

**Figure 4:**
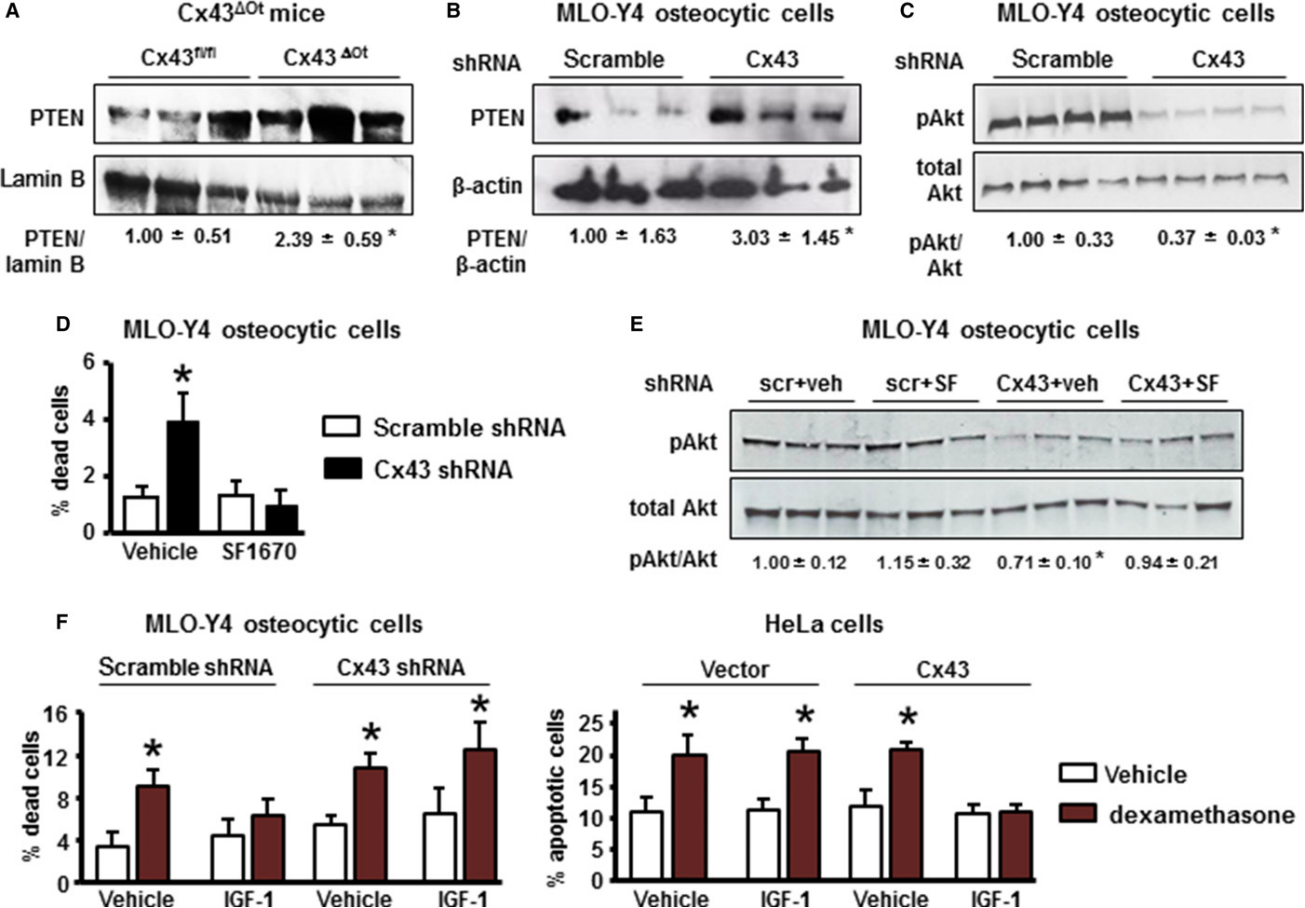
Cx43‐deficiency and aging leads to disruptions in PTEN/pAkt pathway and abolishes the anti‐apoptotic effect of IGF‐1. (A) PTEN expression in ulna from Cx43^fl/fl^ and Cx43^ΔOt^ mice at 4.5 months of age assessed by Western blotting and normalized by lamin B (*n* = 3). *P < 0.05 vs. Cx43^fl/fl^ mice, by *t*‐test. Bars represent mean ± SD. (B,C) PTEN and phosphorylated Akt (pAkt) were analyzed in MLO‐Y4 osteocytic cell lysates by Western blotting (*n* = 3–4). *P < 0.05 vs. corresponding scramble shRNA cells, by *t*‐test. (D) MLO‐Y4 osteocytic cells were treated with vehicle or the PTEN inhibitor SF1670 for 30 min, the media were changed, and the cells were cultured for additional 24 h. Percentage of dead cells was assessed by Trypan blue uptake (*n* = 3). *P < 0.05 vs. corresponding scramble shRNA cells by two‐way ANOVA. (E) pAkt and total Akt were analyzed by Western blotting (*n* = 3). *P < 0.05 vs. corresponding scramble shRNA cells, by *t*‐test. (F) MLO‐Y4 osteocytic cells expressing or not Cx43 and HeLA cells transfected with empty vector or Cx43 together with IGF receptor 1 and nGFP were treated with vehicle or IGF‐1 for 1 h, followed by 6‐h treatment with vehicle or dexamethasone. Cell death was assessed by Trypan blue uptake (for MLO‐Y4 cells) or by nuclear morphology (for HeLa cells) (*n* = 3). *P < 0.05 vs. vehicle‐treated cells for each condition, by two‐way ANOVA.

Consistent with the decrease in miR21 expression, protein levels of PTEN were increased 2.4‐fold in Cx43^ΔOt^ compared to Cx43^fl/fl^ mice (Fig. 4A). Further, reduction of Cx43 expression in MLO‐Y4 osteocytic cells resulted in increased levels of the phosphatase PTEN and decreased phosphorylated Akt, a known target of PTEN (Fig. 4B,C). Cell death was blocked by the PTEN inhibitor SF1670 (Fig. 4D,E), adding support for a role of the phosphatase on cell death induced by Cx43 deficiency.

We next examined whether the pro‐survival effect of IGF‐1, which is mediated by Akt phosphorylation in several cell types, requires Cx43 expression. Because IGF‐1 does not decrease the low level of cell death in vehicle‐treated control cells, we induced apoptosis with the synthetic glucocorticoid dexamethasone, as previously reported (Plotkin *et al*., 2007). IGF‐1 prevented dexamethasone‐induced apoptosis in scramble‐silenced MLO‐Y4 osteocytic cells, and in HeLa cells [which lack endogenous Cx43 (Plotkin *et al*., 2002)] transfected with Cx43, but not in Cx43^def^ cells or in vector‐transfected HeLa cells (Fig. 4F). The levels of dead cells were quantified 7 h after all detached cells were removed from the cultures, unlike the ones shown in previous figures, which were measured at least 24 h after washing the cultures to remove dead cells. Nevertheless, we still detected an increase in the percentage of dead cells in vehicle‐treated Cx43 silenced MLO‐Y4 cells compared to vehicle‐treated scramble shRNA cells (~ 6% vs. ~ 4%).

### Deletion of Cx43 increases the release of pro‐osteoclastogenic cytokines by osteocytic cells

**Figure 5:**
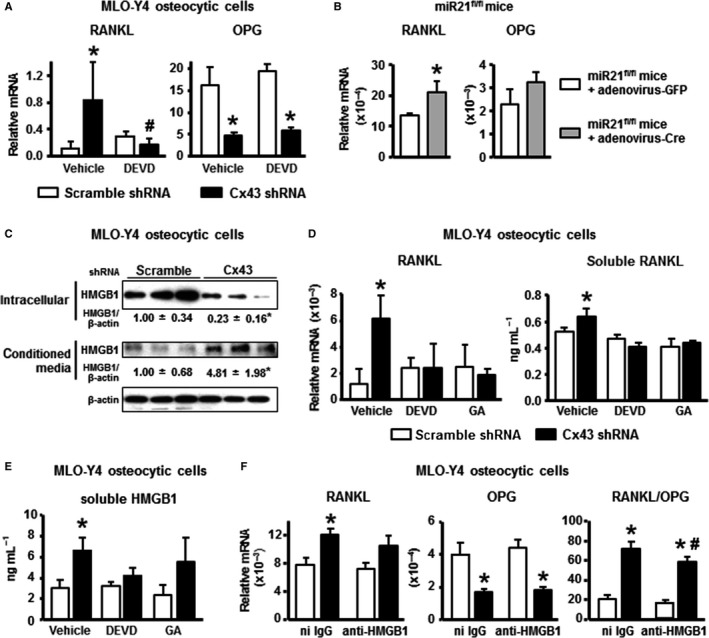
Increased osteocyte apoptosis with reduced Cx43 and miR21 leads to increased RANKL/OPG ratio. (A) Expression of the indicated genes and in MLO‐Y4 cells expressing or lacking Cx43 treated with vehicle or DEVD for 1 h and measured 24 h later. Bars represent mean ± SD (*n* = 6). *P < 0.05 vs. corresponding shRNA scramble cells and ^#^ *P* < 0.05 vs. vehicle‐treated Cx43 shRNA cells by two‐way ANOVA. (B) mRNA levels for the indicated genes in calvaria from miR21^fl/fl^ mice treated with adenovirus‐GFP or adenovirus‐Cre (*n* = 3). Bars represent mean ± SD (*n* = 3). *P < 0.05 vs. adenovirus‐GFP‐treated bone, by *t*‐test. (C) HMGB1 protein expression corrected by β‐actin measured in cell lysate and conditioned medium of MLO‐Y4 osteocytic cells by Western blotting (*n* = 3). (D,E) mRNA levels for RANKL and concentration of sRANKL and HMGB1 in conditioned media from MLO‐Y4 cells expressing or not Cx43, and treated with vehicle, DEVD or GA were measured by qPCR and ELISA, respectively (*n* = 3). (F) Expression of the indicated genes corrected by GAPDH in MLO‐Y4 cells expressing or not Cx43 and treated with nonimmune (ni) IgG or neutralizing anti‐HMGB1 antibodies for 24 h (*n* = 6). *P < 0.05 vs. corresponding shRNA scramble cells and ^#^ *P* < 0.05 vs. ni IgG‐treated Cx43 shRNA cells by two‐way ANOVA.

We next examined the molecular basis for osteoclast recruitment induced by Cx43 deficiency. As found before (Bivi *et al*., 2012), silencing Cx43 from MLO‐Y4 cells results in increased RANKL and reduced oseoprotegerin (OPG) levels (Fig. 5A). Similar results were found in old mice (Cao *et al*., 2003; Almeida *et al*., 2007a). Inhibition of apoptosis with DEVD reversed the increase in RANKL mRNA levels, but did not change OPG mRNA levels in Cx43^def^ cells. Furthermore, removal of miR21 from bones increased RANKL, but did not alter OPG mRNA levels (Fig. 5B). Taken together, these pieces of evidence suggest that the increase in RANKL but not the reduction in OPG levels in the absence of Cx43 is due to increased osteocyte apoptosis downstream of miR21 downregulation.

HMGB1, a ubiquitously expressed molecule, has been shown to increase in the circulation in rats with aging (Fonken *et al*., 2016; Terrando *et al*., 2016) and to increase RANKL expression in osteocytic cells (Yang *et al*., 2008). However, we did not find changes in HMGB1 levels in the serum of old mice (Fig. S2). Silencing of Cx43 in MLO‐Y4 osteocytic cells resulted in a 77% decrease in the amount of intracellular HMGB1, and a 480% increase in the levels of the cytokine in the conditioned media, suggesting increased release of HMGB1 (Fig. 5C). We also detected a tendency toward HMGB1 increase in serum from Cx43^ΔOt^ mice, although it did not reach significance (Fig. S2).

Inhibition of apoptosis with DEVD or blockage of HMGB1 activity with glycyrrhizic acid (GA) reversed the increase in RANKL mRNA levels, and the release of RANKL to the extracellular media (Fig. 5D). In addition, the levels of HMGB1 in conditioned media were reversed to control levels in cells treated with DEVD and attenuated in cells treated with GA (Fig. 5E). GA binds to HMGB1 and blocks HMGB1 chemoattractant and mitogenic activities (Mollica *et al*., 2007) and, unlike its analog glycyrrhetinic acid, does not inhibit connexin channel activity in MLO‐Y4 osteocytic cells (Plotkin *et al*., 2002). Addition of a neutralizing anti‐HMGB1 antibody to Cx43‐silenced MLO‐Y4 cells partially reversed the increase in RANKL mRNA without affecting OPG levels, resulting in a significant decrease in RANKL/OPG ratio, compared to cells treated with nonimmune IgG (Fig. 5F).

### Increased osteoclastogenesis is inhibited by blocking apoptosis of Cx43‐deficient MLO‐Y4 osteocytic cells

**Figure 6:**
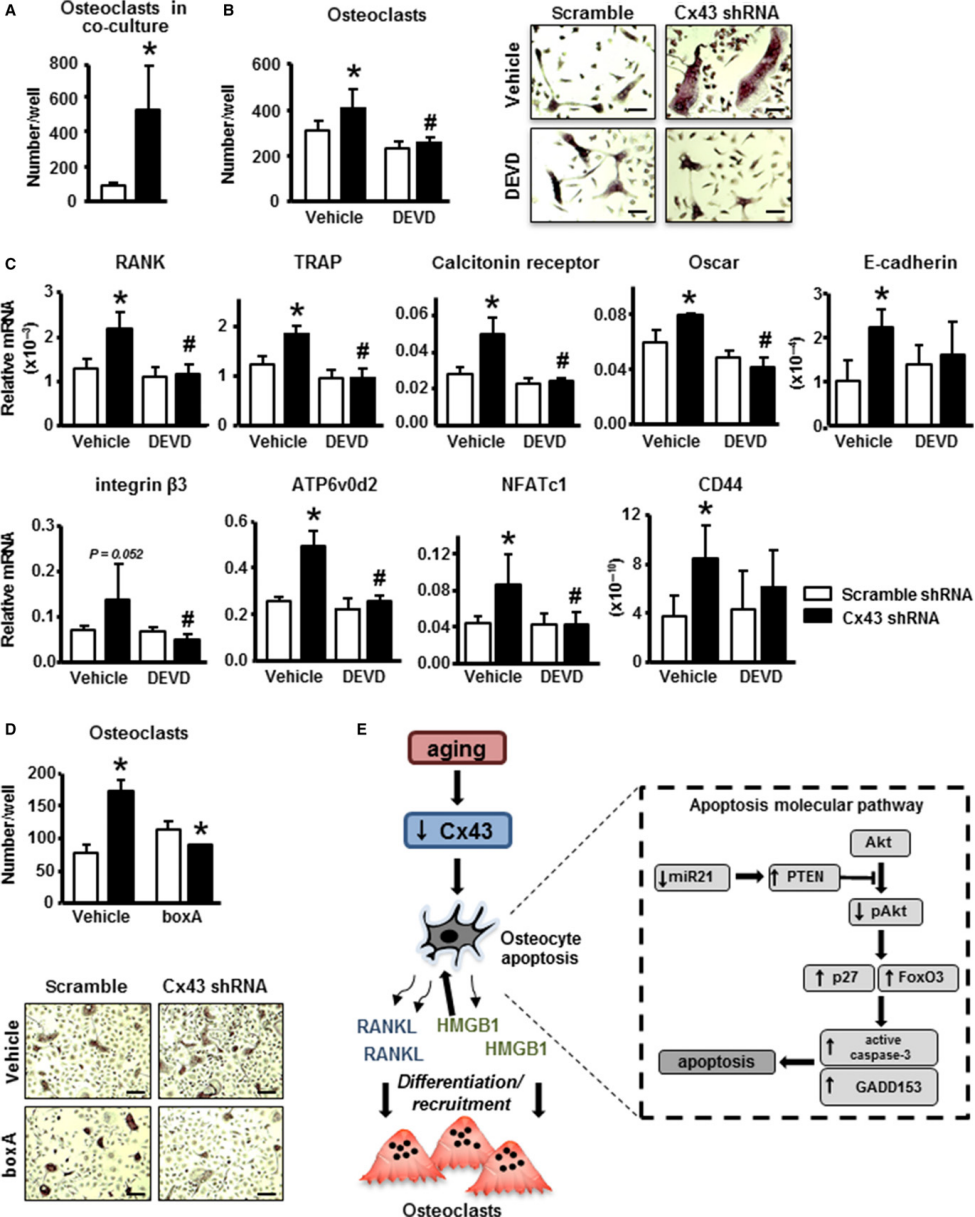
Blockage of apoptosis in Cx43‐silenced MLO‐Y4 osteocytic cells reduces their osteoclastogenic potential. (A) Bone marrow cells isolated from C57Bl/6 mice and co‐cultured with MLO‐Y4 osteocytic cells expressing or not Cx43 for 7 days. Multinucleated (≥ 3 nuclei) osteoclasts were enumerated. Bars represent mean ± SD (*n* = 4). *P < 0.05 vs. shRNA scramble cells by *t*‐test. (B,C) Bone marrow cells were cultured in the presence of conditioned media from MLO‐Y4 osteocytic cells expressing or not Cx43 and treated for 1 h with vehicle or DEVD. (B) Multinucleated osteoclasts and representative images of cultured osteoclasts stained by TRAP and (C) mRNA expression for the indicated genes assessed by qPCR were measured after 7‐day culture. Bars represent mean ± SD (*n* = 4). *P < 0.05 vs. vehicle‐treated shRNA scramble cells, and ^#^ *P* < 0.05 vs. vehicle‐treated Cx43 shRNA cells, by two‐way ANOVA. Scale bars indicate 200 μm. (D) Multinucleated osteoclasts were enumerated after treatment of nonadherent bone marrow cells with conditioned media from MLO‐Y4 osteocytic cells expressing or not Cx43 in the presence of vehicle or boxA. Bars represent mean ± SD (*n* = 4). *P < 0.05 vs. corresponding shRNA scramble cells, by two‐way ANOVA. Representative images of cultured osteoclasts stained by TRAP are shown. Scale bars indicate 100 μm. (E) Working model showing decreased osteocytic Cx43 leads to a reduction in miR21 levels which, in turn, leads to an increase in PTEN levels and consequently a reduction in Akt activation. Decreased Akt activity leads to increased expression of the apoptosis‐related genes p27, FoxO3, and GADD153 resulting in caspase‐3‐mediated apoptosis. At the same time, apoptotic osteocytes release RANKL and HMGB1, which induce osteoclast differentiation and recruitment.

We next investigated whether deletion of Cx43 in osteocytic cells resulted in enhanced osteoclastogenic potential. Increased number of multinucleated TRAP‐positive osteoclasts was observed in co‐cultures of nonadherent bone marrow cells (osteoclast precursors) with Cx43‐silenced MLO‐Y4 cells (Fig. 6A). In addition, conditioned media from cells silenced for Cx43 induced more osteoclasts than media from control cells (Fig. 6B), indicating that factors secreted by the osteocytic cells are responsible for the increased osteoclast differentiation. Inhibition of apoptosis of Cx43^def^ cells with DEVD reduced the osteoclastogenic potential of the conditioned media, which was now similar to conditioned media from scramble shRNA‐treated cells. Furthermore, the expression of osteoclast markers was increased in bone marrow cells treated with conditioned media from Cx43‐deficient cells, whereas their levels were similar to controls when the conditioned media was obtained from cells treated with DEVD (Fig. 6C). Treatment with boxA, which antagonizes HMGB1 binding to its receptor RAGE (Yang *et al*., 2004), decreased osteoclast formation induced by conditioned media from Cx43‐deficient cells (Fig. 6D), suggesting the HMGB1 released by osteocytic cells is responsible for the increase in osteoclast differentiation via activating RAGE.

## Discussion

In the current study, we dissected the molecular signaling pathway underlying osteocyte apoptosis triggered in the absence of osteocytic Cx43 and by aging (Fig. 6E). In addition, we unveiled the molecular link between osteocyte apoptosis and targeted osteoclastic bone resorption. Based on our evidence, we propose that old age and absence of osteocytic Cx43 cause decreased miR21 levels, and the associated increased PTEN protein levels and decreased Akt activation, resulting in osteocyte apoptosis. Apoptotic osteocytes release more HMGB1, which in turn activates RAGE in osteoclast precursors to induce osteoclast differentiation.

Similar to the current report, immunohistochemistry studies showed that Cx43 expression in osteocytes decreases in old mice (Kar *et al*., 2013). On the other hand, the expression of Cx43 is not decreased in osteoblastic cells isolated from old rats (Genetos *et al*., 2012). This evidence suggests that Cx43 expression decreases in osteocytes but not in osteoblasts with aging.

Deletion of Cx43 from osteocytes in mice and stable silencing of Cx43 in MLO‐Y4 osteocytic cells results in spontaneous cell death by a caspase‐3‐dependent mechanism, demonstrating the requirement of Cx43 in the survival of osteocytes [Bivi *et al*. (2012) and this report]. Unlike the studies in mice lacking Cx43 (Bivi *et al*., 2012; Lloyd *et al*., 2013), ubiquitous expression of a mutated Cx43 (G60S/+ mice) does not result in increased osteocyte apoptosis, changes in osteocyte number or altered number of empty lacunae (Zappitelli *et al*., 2013). This evidence suggests that the level and activity of Cx43 in the Cx43 G60S/+ mice is sufficient to maintain osteocyte viability and that the phenotype of the G60S/+ mice is likely due to the role of Cx43 in early osteoblastic cells and their progenitors.

The survival effect of Cx43 does not involve the scaffold or regulatory domains present in Cx43 C‐terminus. This is consistent with our previous study showing that expression of a truncated Cx43 lacking the C‐terminus domain restores osteocyte viability in mice lacking Cx43 in osteocytes (Pacheco‐Costa *et al*., 2015). Similarly, the altered bone material mechanical properties, with decreased stiffness, of mice lacking osteocytic Cx43 are reversed by the truncated Cx43. Nevertheless, our *in vitro* data, together with our previous *in vivo* study (Pacheco‐Costa *et al*., 2015) and a recent report in male mice expressing only the truncated Cx43 (Moorer *et al*., 2017), suggest that the trans‐membrane and cytoplasmic amino‐terminal domains are sufficient to maintain osteocyte viability and preserve normal cortical bone geometry and material properties. Future studies using mice overexpressing Cx43 in osteocytes (currently under development in our laboratory) will allow us to determine whether maintenance of Cx43 levels reverses the increased osteocyte apoptosis and, at least partially, the skeletal phenotype of old mice.

The underlying molecular mechanisms that lead to increased osteocyte apoptosis in the absence of Cx43 and in old age remained unclear. We now propose the existence of a signaling cascade that maintains cell viability through the regulation of miR21 downstream of Cx43 (Fig. 6E). Although we also found that miR218 levels are increased in Cx43^def^ cells, this increase was not reproduced in bones from old mice or mice lacking Cx43 in osteocytes, suggesting that whereas miR21 is regulated by Cx43, signals other than Cx43 are involved in miR218 regulation *in vivo*.

Several pieces of evidence support the role of miR21 on osteocyte apoptosis. First, both Cx43^ΔOt^ mice and Cx43‐deficient osteocytic cells exhibit accelerated cell death and express lower levels of miR21, a pro‐survival miR (Garzon *et al*., 2009). Further, miR21 deletion by adenovirus‐Cre in miR21^fl/fl^ calvaria bones increases the expression of apoptosis‐related genes; and reducing miR21 expression using oligonucleotide inhibitors induces apoptosis of control osteocytic cells. Conversely, a miR21 oligonucleotide mimic attenuates Cx43‐deficient osteocyte apoptosis, supporting the idea that miR21 reduction causes the increased osteocyte apoptosis in the absence of Cx43. Consistently, the expression of both Cx43 and miR21 is reduced in old bones, suggesting that the decreases in Cx43 and miR21 levels contribute to the decreased osteocyte viability in old age. Although the mechanism by which reduced Cx43 expression leads to decreases in miR21 levels is not known, these two genes are not located in the same gene locus (Kozomara & Griffiths‐Jones, 2014), excluding the possibility that a deletion of one gene removes the other one.

In addition to inducing apoptosis, miR21 deletion increases RANKL expression. The regulation of RANKL expression in Cx43‐deficient osteocytes could be a consequence of increased apoptosis in the absence of miR21, or to the lack of direct actions of miR21 on the RANKL gene. However, miR21 deletion reduces RANKL expression in multiple myeloma‐exposed bone marrow cells (Pitari *et al*., 2015), suggesting that direct actions of the miR on gene expression are not the cause of increased RANKL levels in our study. It is therefore likely that the changes in RANKL expression in cells in which miR21 was deleted are a consequence of increased apoptosis. Future studies are needed to further support this conclusion.

The mechanism by which old age leads to increased osteocyte apoptosis is not completely understood. Previous studies showed that the survival effect of IGF‐1 is reduced in osteoblastic cells isolated from old (24‐month‐old) compared to young (1.5‐month‐old) and adult (6‐month‐old) mice, even though the levels of the IGF‐1 receptor are increased in the older mice (Cao *et al*., 2007). The resistance of bone marrow cells from old mice to IGF‐1 effect was associated with reduced activation of Akt, a known mediator of survival induced by the cytokine (Ma *et al*., 2011). We now propose that reduced response to IGF‐1 in old animals is due to decreased Cx43/miR21 levels, counteracting the effect of IGF‐1 on the PTEN/pAkt pathway. Consistent with this notion, IGF‐1 prevents apoptosis of osteoblastic cells (Hill *et al*., 1997; Grey *et al*., 2003) and Cx43 is required for the survival effect of IGF‐1 in cardiomyocyte precursors (Lu *et al*., 2009).

Osteocyte apoptosis has been shown to precede and to be associated with increased targeted recruitment of osteoclasts along adjacent bone surfaces in several animal models [recently reviewed by us (Plotkin, 2014; Plotkin & Bellido, 2016)]. Consistently, we showed that osteocyte‐specific Cx43 deletion leads to accumulation of apoptotic osteocytes, and an associated increase in osteoclasts in some adjacent areas on endocortical bone surfaces (Bivi *et al*., 2012). Additionally, Cx43‐deficient osteocytic cells exhibit an increase in the RANKL/OPG ratio, facilitating osteoclast formation. However, increased populations of osteocytes with reduced OPG levels are present even in areas lacking osteoclasts in Cx43^ΔOt^ mice, suggesting that apoptotic osteocytes release other signals that are required for osteoclast recruitment. The specific molecules underlying this increased osteoclast recruitment were heretofore unknown. We now show that Cx43‐deficient osteocytes release elevated levels of the pro‐inflammatory cytokine HMGB1. Similarly, induction of MLO‐Y4 osteocytic cell apoptosis with TNF‐α/cycloheximide increases HMGB1 release (Yang *et al*., 2008). However, in Yang's study, addition of HMGB1 to the cells increased Akt phosphorylation, rather than decreased it, as reported here. This evidence suggests that the changes in Akt activity in the Cx43‐deficient cells are not a response to the released HMGB1, but the result of the increase in PTEN levels downstream of miR21 downregulation.

We also found that inhibition of osteocyte apoptosis with DEVD or treatment with a HMGB1 neutralizing antibody or GA, an inhibitor of HMGB1 action (Zhang *et al*., 2015), reduces RANKL expression in Cx43‐deficient cells. This is consistent with previous work showing that apoptotic MLO‐Y4 osteocytic cells release HMGB1 (Charoonpatrapong *et al*., 2006; Klune *et al*., 2008). Further, HMGB1 is chemotactic for osteoclasts and triggers osteoclastogenesis by activating RAGE (Taniguchi *et al*., 2007; Zhou *et al*., 2008; Yang *et al*., 2013). Consistently, inhibition of apoptosis prevents RANKL and HMGB1 release and attenuates the enhanced osteoclastogenesis and osteoclast marker expression induced by conditioned media from Cx43‐deficient osteocytes. In addition, the HMGB1‐RAGE receptor antagonist boxA (Yang *et al*., 2004) blocks osteoclastogenesis induced by conditioned media from Cx43‐deficient osteocytes. However, we could not detect systemic changes in HMGB1 levels in Cx43‐deficient or old mice. Therefore, our results suggest that HMGB1 released by Cx43‐deficient osteocytes in their vicinity enhances osteoclastogenesis by increasing RANKL in osteocytes and, at the same time, directly stimulating osteoclast precursor differentiation at least partially through activation of RAGE. Future studies will allow us to further elucidate the mechanisms by which apoptotic osteocytes in Cx43^ΔOt^ and old mice release HMGB1 and the role of the RAGE and TLR4 receptors on the induction of osteoclast recruitment.

In summary, we propose that gap junction communications through Cx43 channels maintain osteocyte viability via downstream regulation of miR21, leading to the subsequent inhibition of PTEN activity and preservation of the Akt survival pathway (Fig. 6E). Additionally, our findings support a model where reduced Cx43 levels in osteocytes in old animals lead to apoptosis and the release of RANKL and HMGB1, which signal osteoclasts to increase resorption along associated bone surfaces. We therefore identified a novel Cx43/miR21/HMGB1/RANKL pathway mediated by gap junction communications in osteocytes that could be targeted to treat bone fragility with aging.

## Experimental procedures

### Mice

C57BL/6 female mice were purchased from National Institute on Aging and sacrificed at 4, 12, 18, 21, and 24 months of age. To delete Cx43 specifically in osteocytes, mice were generated using the Cre/LoxP system (Bivi *et al*., 2012). Cx43^fl/fl^ mice were crossed with mice harboring DMP1‐8 kb‐Cre, which express Cre‐recombinase under control of a DNA fragment containing 8 kb of the murine dentin matrix protein 1 promoter (named Cx43^ΔOt^). The mouse strain with engineered lox cassettes on both sides of the mmu‐miR‐21 genomic locus (named miR21^fl/fl^) was generated by Dr. Ivan through Ozgene Pty Ltd, a service provider of genetically modified mice (Perth, Australia). All mice used were maintained in a C57BL/6 background. The protocols involving mice were approved by the Institutional Animal Care and Use Committee of Indiana University School of Medicine.

### Cell culture, silencing, and transient transfections

MLO‐Y4 osteocytic and Ob‐6 osteoblastic cells were silenced using short hairpin (sh)RNA Lentiviral Particles (Sigma‐Aldrich Chemical Co., St. Louis, MO, USA), as previously reported (Plotkin *et al*., 2008; Bivi *et al*., 2011), and cultured as previously described. The efficiency of deletion was determined by quantifying Cx43 protein and mRNA levels by Western blotting and by qPCR, respectively. All samples from *in vitro* studies were collected 24 h after seeding the cells or 48 h after transfection, unless otherwise indicated. The plasmid encoding the full‐length rat Cx43 (abbreviated as Wt) was provided by R. Civitelli (Washington University, Saint Louis, MO) (Lecanda *et al*., 1998). The mutant Cx43 truncated at amino acid 245 (abbreviated as Cx43^Δ245^) (Zhou *et al*., 1999) and the Cx43 carboxy‐terminal tail (abbreviated as Cx43^C‐tail^) (Zhou *et al*., 1999) were provided by B. Nicholson (University of Texas, Santo Antonio, TX). Cx43 lacking seven residues from the internal loop at positions 130–136 (abbreviated as Cx43^Δ130^) was provided by V.A. Krutovskikh (International Agency for Research on Cancer, Lyon, France) (Krutovskikh *et al*., 1998). Cx43^Cys‐less^, which has all cysteine sites on the two extracellular loops replaced by alanine (abbreviated as Cx43^Cys‐less^), was provided by G.M. Kidder (University of Western Ontario, Ontario, Canada) (Tong *et al*., 2007). All of the constructs used in this study are of rat origin and have been shown to produce functional proteins. pcDNA3.1 empty vector was used as control (Invitrogen, Grand Island, NY, USA). MLO‐Y4 osteocytic cells were transiently transfected with different DNA constructs together with nuclear GFP using Lipofectamine Plus (Invitrogen) reagent with 0.1 μg cm^−2^ as described previously (Bivi *et al*., 2011). Apoptosis was assessed 48 h after transfection by quantification of cells exhibiting nuclear fragmentation and chromatin condensation under an EVOS fluorescence microscope system (Life Technologies, Carlsbad, CA, USA).

HeLa cells (2 × 10^4^cells cm^−2^) were transiently transfected with either empty vector or rat Cx43 together with IGFR1 and nuclear GFP using Lipofectamine Plus, as published (Plotkin *et al*., 2002), and apoptosis was assessed as indicated below.

### Western blotting analysis

Whole protein extracts from MLO‐Y4 osteocytic cells or from bone were prepared as published (Plotkin *et al*., 2011; Bivi *et al*., 2012). To determine the levels of HMGB1 on culture supernatants, proteins were concentrated 10× by precipitation using trichloroacetic acid (5:1). Protein lysates were separated on 10% SDS–PAGE gels and electrotransferred to polyvinylidene difluoride membranes (Millipore, Billerica, MA, USA). These membranes were incubated in blocking solution (5% nonfat milk) for 30 min and probed with primary antibodies diluted 1:1000 in 5% nonfat milk against a monoclonal anti‐PTEN (Sc‐7974), polyclonal anti‐GADD153 (Sc‐575), anti‐lamin B (Sc‐6217) (Santa Cruz Biotechnology, Santa Cruz, CA, USA), monoclonal anti‐Akt (2920), anti‐phosphorylated Akt at serine 473(4060) (Cell Signaling Technology, Danvers, MA, USA), anti‐cleaved caspase‐3 (Asp175) (PA5‐23921; Thermo Fisher Scientific, Rockford, IL, USA), anti‐connexin43 (C6219) and anti‐β‐actin (A5316) (Sigma‐Aldrich), and anti‐HMGB1 (ab18256) (Abcam, Cambridge, MA, USA) overnight at 4 °C, followed by corresponding secondary antibodies conjugated with horseradish peroxidase in 5% nonfat milk (Santa Cruz Biotechnology) for 4 h at room temperature. After rinsing with TBS‐T, the membranes were developed with an enhanced chemiluminescence Western blotting substrate kit (Pierce Biotechnology Inc., Rockford, IL, USA). Bands were detected, and their intensity was quantified using the TotalLab TL 100 software (Nonlinear Dynamics Ltd., Durham, NC, USA).

### RNA extraction and real‐time PCR (qPCR)

Total RNA was isolated and purified using TRIzol (Invitrogen), as published (Pacheco‐Costa *et al*., 2014). Reverse transcription was performed using a high‐capacity cDNA kit (Applied Biosystems, Foster City, CA, USA). qPCR was performed using the Gene Expression Assay Mix TaqMan Universal Master Mix with an ABI 7900HT real‐time PCR system. The housekeeping gene glyceraldehyde 3‐phosphate dehydrogenase (GAPDH) was used. Primers and probes were commercially available (Applied Biosystems) or were designed using the Assay Design Center (Roche Applied Science, Indianapolis, IN, USA). To evaluate miRNA's expression level, total RNA was isolated and purified using TRIzol reagent and 10 ng was reversely transcribed to cDNA using TaqMan miR reverse transcription kit (Applied Biosystems, Carlsbad, CA, USA) to detect and quantify mature miR21 (assay ID: 000397), miR18 (assay ID: 000521), and miR135 (assay ID: 001230). The reaction mixtures were incubated at 16 °C for 30 min, 42 °C for 30 min, 85 °C for 5 min, and 4 °C for 30 s. The values were normalized for housekeeping miR135. Relative expression was calculated using the ∆Ct method.

### IGF‐1, DEVD, and PTEN inhibitor treatment

MLO‐Y4 osteocytic cells or HeLa cells were treated with vehicle or 5 ng mL^−1^ IGF‐1 for 1 h, followed by 6‐h treatment with vehicle or 10^−6^ m dexamethasone. In addition, in a parallel experiment, MLO‐Y4 osteocytic cells were treated with vehicle or 1 μm SF1670 (Abcam Biochemical, Cambridge,MA, USA.) for 30 min and then cultured for 24 h. For apoptosis inhibition, cells were treated for 1 h with 50 nm Ac‐DEVD‐CHO (DEVD, Biotium, Inc., Hayward, CA, USA), a caspase‐3/7 inhibitor, which was then removed and cells were cultured in fresh media for 24 h, or treated for 24 h with 50 nm DEVD. To determine the percentage of dead cells, supernatant was removed and cells were re‐suspended in 25 μL of 1× Trypan blue. Ten microliters of the cell suspension were transferred to a hemocytometer, and the number of viable cells (clear, transparent) and dead (blue) was counted. Data are reported as percentage of dead cells. Cells were transfected with the indicated constructs together with nuclear GFP to assess the nuclear morphology only in transfected cells. Forty‐eight hours after transfection cells were washed to remove dead cells, and treated with 5 ng mL^−1^ IGF‐1 for 1 h, followed by 10^−6^ m dexamethasone. Cells were fixed 6 h later, and the prevalence of apoptotic cells exhibiting chromatin condensation and nuclear fragmentation was assessed under fluorescence microscopy. Data are reported as percentage of apoptotic cells.

### miR array analysis

The effect of Cx43 silencing on miRNA levels in MLO‐Y4 osteocytic cells was assessed using the Apoptosis‐Associated miRNA Plate Array, as described by the manufacturer (Signosis Inc., Santa Clara, CA, USA; cat. # MA‐1002). The values were normalized for U6 RNA.

### miR silencing and overexpression

MLO‐Y4 osteocytic cells silenced or not for Cx43 were plated at the density of 2 × 10^4 ^cells cm^−2^ on 48‐well plates coated with type I rat tail collagen and cultured overnight. Cells were transiently transfected using Lipofectamine RNAiMAX reagent (Invitrogen) containing miRIDIAN negative control inhibitor, miR21 inhibitor, negative control mimic, or miR21 mimic (GE Healthcare Bio‐Sciences, Pittsburgh, PA, USA) at a final concentration of 0.1 nm in medium without serum and penicillin/streptomycin for 6 h. Next, medium 2× concentrated was added to each well and then cultured overnight. Medium was changed to a regular growing medium, and cells were then cultured for an additional 24 h before measuring cell death by Trypan blue uptake. miR21 levels were decreased by 60% in MLO‐Y4 scramble cells and by 91% in MLO‐Y4 Cx43 shRNA cells treated with miR21 inhibitor, as measured by qPCR. miR21 levels were increased by 44% in MLO‐Y4 scramble cells and 234% in MLO‐Y4 Cx43 shRNA cells treated with the miR21 mimic.

### Viral infection of calvaria bone *ex vivo*

Calvariae of miR21^fl/fl^ mice were harvested at 5 days of age, and two 5‐mm pieces of the bone were removed using a biopsy punch (Integra LifeSciences Corporation, Plainsboro, NJ, USA). Samples were washed in PBS and incubated in a 96‐well plate with α‐minimal essential medium supplemented with 10% FBS and 1% penicillin/streptomycin for 6 h. Bones were then treated with 2.5 μL per well of control adenovirus (Adeno‐GFP, cat.#1060) or Cre‐recombinase virus (cat.#1700) (Vector BioLabs, Malvern, PA, USA) diluted in serum‐free α‐minimal essential medium in a final volume of 45 μL overnight. Next, media were removed and samples were washed twice with PBS before adding α‐minimal essential medium supplemented with 10% FBS and 1% penicillin/streptomycin and cultured for additional 48 h at 37% and 5% CO_2_. mRNA was quantified by qPCR and protein quantified by Western blotting.

### Transmission electron microscopy (TEM)

Calvaria bones from miR21^fl/fl^ mice treated with adenovirus (Adeno‐GFP, cat.#1060) or Cre‐recombinase virus were processed for TEM as previously published (Bivi *et al*., 2012). Briefly, bones were decalcified and postfixed in 2% paraformaldehyde/2% glutaraldehyde in 0.1 m cacodylate buffer for 1 h, followed by 1 h‐treatment with 1% osmium tetroxide in 0.1 m cacodylate buffer. After standard dehydration and embedding in Embed 812 (Electron Microscopy Sciences, Hatfield, PA, USA), the blocks were sectioned at 85 nm and placed on copper grids, stained with uranyl acetate, and viewed on a Tecnai G2 12 Bio Twin electron microscope (FEI, Hillsboro, OR, USA) at the Electron Microscopy Center of the Department of Anatomy and Cell Biology (Indiana University School of Medicine). Digital images were taken with an Advanced Microscope Techniques (Danvers, MA, USA) CCD camera.

### Osteoclastogenesis in co‐cultures and with conditioned medium

Bone marrow cells were isolated from C57Bl/6 mice by flushing the bone marrow out with α‐minimal essential medium supplemented with 10% FBS and 1% penicillin/streptomycin and cultured for 24–48 h. Next, nonadherent were collected and 2 × 10^5^ cells cm^−2^ were seeded on top of MLO‐Y4 osteocytic cells silenced or not for Cx43. Cells were cultured in the presence of 10 nm 1.25(OH)_2_ vitamin D3 and 1 μm PGE2. Medium was changed every 2 days for 5 days, as previously published (Miyazaki *et al*., 2003). The conditioned medium from MLO‐Y4 osteocytic cells silenced or not for Cx43 was collected after 24 h‐culture and then concentrated 4× using centricon filters (10 kDa cutoff) (EDM Millipore). Media was diluted 1:4 and added to 48‐well plate containing 2 × 10^5^ nonadherent cells cm^−2^ with 40 ng mL^−1^ RANKL and 20 ng mL^−1^ M‐CSF (Pacheco‐Costa *et al*., 2014). Growing medium that had not been in contact with the cells was used as control. Osteoclasts exhibiting 3 or more nuclei were enumerated after staining for TRAPase using a commercial kit (Sigma‐Aldrich). Images were acquired using a Zeiss Axiovert 35 microscope equipped with a digital camera Carl Zeiss Microimaging Inc., Thronwood, NY, USA.

### Neutralization of HMGB1 in cell cultures

MLO‐Y4 osteocytic cells silenced or not for Cx43 were plated at the density of 2 × 10^5 ^cells cm^−2^ on six‐well plates coated with type I rat tail collagen and cultured overnight. Cells were either treated with 1 mg mL^−1^ GA (Zhang *et al*., 2015) or with 0.5 μg mL^−1^ nonimmune (ni) rabbit IgG (Abcam, cat.# ab171870) or neutralizing rabbit anti‐HMGB1 (Abcam, cat.# ab18256) antibodies for 24 h and then concentrated 4× using centricon filters (10 kDa cut off) (EDM Millipore). For cultures with immunoglobulins, conditioned media were incubated with 10 μL mL^−1^ protein A agarose (Roche Applied Science, cat.# 11719408001) overnight at 4 °C to remove the immunoglobulins. One molar HEPES was then added to IgG‐depleted conditioning media, which was then aliquoted and stored at 80 °C until used.

### Soluble RANKL, OPG, and HMGB1 levels in conditioned media

Conditioned media from MLO‐Y4 cells were collected, aliquoted, and stored at −80 °C until used. RANKL, OPG, and HMGB1 protein levels in the supernatants were determined using Quantikine Mouse RANKL Immunoassay (R&D Systems, Inc., Minneapolis, MN, USA, cat.# MTR00), Quantikine Osteoprotegerin Immusoassay (R&D Systems, Inc., cat.# MOP00), or HMGB1 Immunoassay (IBL International, Hamburg, Germany, cat.# ST51011), respectively.

### Statistical analysis

Data were analyzed using SigmaPlot (Systat Software Inc., San Jose, CA, USA). All values are reported as the mean ± standard deviation. Differences were evaluated either by one‐ or two‐way ANOVA, with *post hoc* analysis using Tukey Method or by Student's *t*‐test, as appropriate. Differences were considered significant when *P* < 0.05.

Additional methods are included in Data S1 under supplementary material.

## Author contribution

Study design was performed by HMD, RPC, and LIP. Data acquisition was performed by HMD, RPC, EGA, LRB, ARG, JH, MH, SAB, AB, and LIP. Advice on experimental design and contribution of materials/animals was performed by TY and MI. Data analysis and interpretation was performed by RPC, HMD, TB and LIP. Drafting of manuscript was performed by HMD, RPC, and LIP. All authors revised the manuscript and approved the final version.

## Funding

This research was supported by the National Institutes of Health (R01‐AR067210 and R01‐AR053643) to LIP and R01‐CA155332 to MI. RPC received a scholarship from Coordination of Improvement of Higher Level Personnel (CAPES), Brazil (PDEE: #1065/11‐4). EGA was supported by Life‐Health Sciences Internship Program and the CTSI summer scholars program at IUPUI. JH received a scholarship from Women in Science Summer Internship with the Indiana BioMedical Gateway Program, IUSM. BAS was supported by the NIH‐NHLBI T35 HL110854‐01 grant. LRB was supported by a grant from the Universidad Nacional de Rosario, Rosario, Argentina.

## Conflict of interest

The authors have no conflict of interest to declare.

## Supporting information


**Fig. S1** Deletion of Cx43 does not affect Ob‐6 cells but leads to caspase3‐mediated apoptosis in MLO‐Y4 osteocytic cells.

**Fig. S2** HMGB1 levels are not altered systemically with aging or in osteocytic Cx43‐defient mice.

**Data S1** Methods.
