# Supplementary figures and images for: Disruption of the Cx43/miR21 pathway leads to osteocyte apoptosis and increased osteoclastogenesis with aging
Source: Aging Cell. 2017 Mar 19;16(3):551–63. doi: 10.1111/acel.12586 (PMC5418188; doi:10.1111/acel.12586)

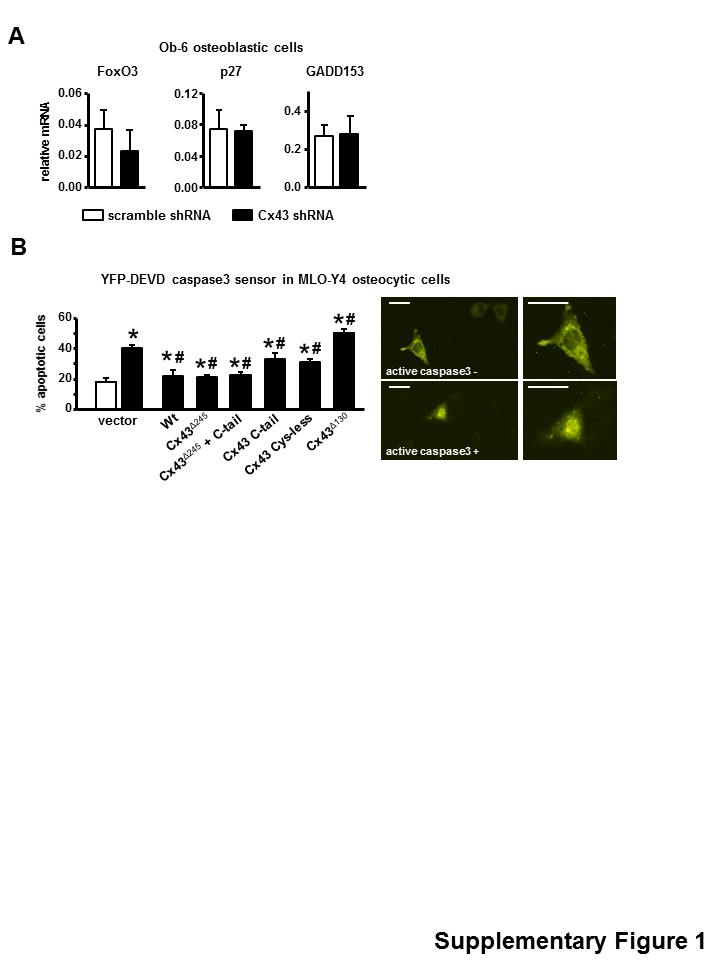

Supplement: Supplementary file 1 — Fig. S1 Deletion of Cx43 does not affect Ob‐6 cells but leads to caspase3‐mediated apoptosis in MLO‐Y4 osteocytic cells. [file ACEL-16-551-s001.TIF]

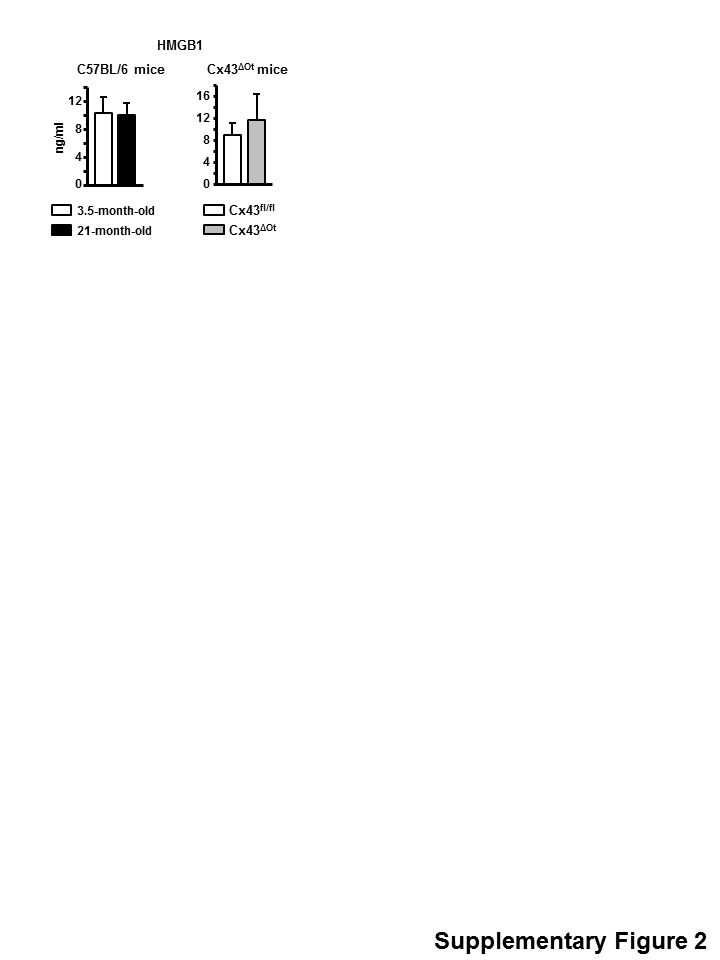

Supplement: Supplementary file 2 — Fig. S2 HMGB1 levels are not altered systemically with aging or in osteocytic Cx43‐defient mice. [file ACEL-16-551-s002.TIF]
