# Supplementary material for: Disruption of the Cx43/miR21 pathway leads to osteocyte apoptosis and increased osteoclastogenesis with aging
Source: Aging Cell. 2017 Mar 19;16(3):551–63. doi: 10.1111/acel.12586 (PMC5418188; doi:10.1111/acel.12586)
Supplement: Supplementary file 3 — Data S1 Methods. [file ACEL-16-551-s003.docx]

**SUPPLEMENTARY FIGURE LEGEND**

**Suppl. Fig. 1. Deletion of Cx43 does not affect Ob-6 cells but leads to caspase3-mediated apoptosis in MLO-Y4 osteocytic cells. (A)** mRNA levels were measured in Ob-6 cells expressing (scramble) or lacking (shRNA) cx43 by qPCR and corrected by GAPDH. Bars represent mean ± S.D. (n=3). *p<0.05 versus scramble cells, by t-test. **(B)** MLO-Y4 osteocytic cells were transiently transfected with empty vector or the indicated Cx43 constructs together with YFP-DEVD-caspase3 sensor. *p<0.05 versus vector-transfected scramble shRNA cells, ^#^p<0.05 versus vector-transfected Cx43 shRNA cells by one-way ANOVA. Representative images of cells are shown. Scale bars indicate 50µm.

**Suppl. Fig. 2. HMGB1 levels are not altered systemically with aging or in osteocytic Cx43-defient mice.** Serum HMGB1 levels were assessed in C57BL/6 mice (n=8-10) and in osteocytic Cx43-defient mice (n=6-7) by ELISA.

**METHODS**

***YFP-DEVD-caspase 3 sensor***

MLO-Y4 osteocytic cells were transiently transfected with different DNA constructs together with YFP-DEVD-caspase 3 sensor using Lipofectamine Plus (Invitrogen) reagent with 0.1µg/cm^2^ as described previously (Bivi et al., 2011). Apoptosis was assessed by measuring caspase 3 activation in cells transiently transfected with the YFP-DEVD-caspase 3 sensor, as previously reported (Liu et al., 2004; Plotkin et al., 2006; Plotkin et al., 2007). The caspase 3 sensor is composed of YFP bound to the DEVD amino acid sequence cleaved by caspase 3, and also contains a dominant N-terminal nuclear export signal and a C-terminal nuclear localization signal. In living cells YFP is localized in the cytoplasm since caspase 3 is inactive and the nuclear export sequence is maintained. In apoptotic cells, active caspase 3 cleaves off the protein at the DEVD sequence and the YFP caspase 3 sensor is localized to the nucleus. The percentage of cells with activate caspase 3 was determined by quantifying the caspase 3 sensor localization 48h after transfection, using an EVOS fluorescence microscope system (Life Technologies, Carlsbad, CA).

***Serum HMGB1***

Blood was collected by cheek bleeding after 6 hours of fasting. Serum was collected, aliquoted, and stored at −80 °C until used. High mobility group box 1 protein (HMGB1) (IBL International GMBH, Germany, cat.# ST51011) was measured as described by the manufacturer.

**References**

Bivi,N., Lezcano,V., Romanello,M., Bellido,T., and Plotkin,L.I. (2011). Connexin43 interacts with βarrestin: a pre-requisite for osteoblast survival induced by parathyroid hormone. J. Cell. Biochem. *112*, 2920-2930.

Liu,Y., Porta,A., Peng,X., Gengaro,K., Cunningham,E.B., Li,H., Dominguez,L.A., Bellido,T., and Christakos,S. (2004). Prevention of glucocorticoid-induced apoptosis in osteocytes and osteoblasts by calbindin-D28k. J. Bone Miner. Res. *19*, 479-490.

O'Brien,C.A., Plotkin,L.I., Galli,C., Goellner,J., Gortazar,A.R., Allen,M.R., Robling,A.G., Bouxsein,M., Schipani,E., Turner,C.H., Jilka,R.L., Weinstein,R.S., Manolagas,S.C., and Bellido,T. (2008). Control of bone mass and remodeling by PTH receptor signaling in osteocytes. PLoS ONE *3*, e2942.

Plotkin,L.I., Manolagas,S.C., and Bellido,T. (2006). Dissociation of the pro-apoptotic effects of bisphosphonates on osteoclasts from their anti-apoptotic effects on osteoblasts/osteocytes with novel analogs. Bone *39*, 443-452.

Plotkin,L.I., Manolagas,S.C., and Bellido,T. (2007). Glucocorticoids induce osteocyte apoptosis by blocking focal adhesion kinase-mediated survival: evidence for inside-out signaling leading to anoikis. J. Biol. Chem. *282*, 24120-24130.
